# Non-coding RNA suppresses FUS aggregation caused by mechanistic shear stress on pipetting in a sequence-dependent manner

**DOI:** 10.1038/s41598-021-89075-w

**Published:** 2021-05-04

**Authors:** Nesreen Hamad, Ryoma Yoneda, Masatomo So, Riki Kurokawa, Takashi Nagata, Masato Katahira

**Affiliations:** 1 Institute of Advanced Energy, Kyoto University, Kyoto, 611-0011 Japan; 2 Division of Biomedical Sciences, School of Medicine, Saitama Medical University, Saitama, 350-0495 Japan; 3 Institute for Protein Research, Osaka University, Osaka, 565-0871 Japan; 4 Graduate School of Energy Science, Kyoto University, Kyoto, 606-8501 Japan

**Keywords:** Protein aggregation, Long non-coding RNAs, Protein aggregation, Intrinsically disordered proteins, Neurological disorders

## Abstract

Fused in sarcoma/translocated in liposarcoma (FUS/TLS) is a multitasking RNA/DNA binding protein. FUS aggregation is implicated in various neurodegenerative diseases. RNA was suggested to modulate phase transition of FUS. Here, we found that FUS transforms into the amorphous aggregation state as an instant response to the shear stress caused by usual pipetting even at a low FUS concentration, 100 nM. It was revealed that non-coding RNA can suppress the transformation of FUS into aggregates. The suppressive effect of RNA on FUS aggregation is sequence-dependent. These results suggested that the non-coding RNA could be a prospective suppressor of FUS aggregation caused by mechanistic stress in cells. Our finding might pave the way for more research on the role of RNAs as aggregation inhibitors, which could facilitate the development of therapies for neurodegenerative diseases.

## Introduction

Fused in sarcoma/translocated in liposarcoma (FUS/TLS) is an RNA/DNA binding protein, which regulates various biological processes^1–5^. FUS has been considered as a molecular link between apparently different human diseases such as cancer and neurodegenerative diseases^6–11^. FUS was found as the major component of nuclear polyglutamine (polyQ) aggregates in a Huntington disease (HD) cell model^8^, where FUS was converted from a soluble form to insoluble aggregates^9^. Then, FUS was also found to be a member of the PolyQ aggregates in other diseases including spinocerebellar ataxia (SCA) types 1, 2, and 3, and dentatorubral-pallidoluysian atrophy (DRPLA)^7^. Around the same time, FUS mutations were found in amyotrophic lateral sclerosis (ALS)^10,11^ and frontotemporal lobar degeneration (FTLD) patients^12^. Those mutations were found to accelerate the FUS transition into an insoluble form^13^. Although the above-mentioned neurodegenerative diseases have different manifestations, FUS aggregation is associated with all of them^5^, which suggests a common pathway for their neuropathologies.

Dysregulation of RNA metabolism is a major cause of various human diseases^14,15^. The implication of mutations of the RNA-binding domain of FUS in the etiologies of neurodegenerative diseases suggests that the RNA binding ability of FUS is necessary to maintain neuron functionality. Therefore, FUS is considered as an emerging therapeutic target for neurodegenerative diseases as well as cancer prevention and treatment^16^.

FUS is known to take on different states such as dispersed, liquid droplet, gel, and fibril ones depending on factors such as pH, ionic strength, protein concentration, thermal stress, shear stress, and RNA presence^17–20^. It was found that dynamic liquid-like FUS-containing droplets yielded by liquid–liquid phase separation (LLPS) play a key role in the assembly of membrane-less organelles such as stress granules^21^. It was also found that high concentration of RNA can suppress LLPS of FUS^18^. It was noted that in physiological conditions, FUS can interchange between a dispersed phase, liquid droplets, and a reversible gel, while through aging or pathological conditions, liquid droplets can be converted into irreversible gels and fibrils^17,22^.

FUS consists of a low complexity domain (LC domain), three arginine-glycine-glycine-rich domains (RGG domains), an RNA recognition motif (RRM), and a zinc-finger domain (ZnF domain). Only the RRM and ZnF domains are structured, the others being regarded as intrinsically disordered regions (IDRs). Previously, we showed by fluorescence resonance energy transfer (FRET) and high-speed atomic force microscopy (HS-AFM) analyses that FUS takes on a compact conformation in its free-form but becomes extended upon binding to RNA/DNA^23,24^.

In this study we found that mechanistic shear stress caused by pipetting can induce FUS aggregation by means of fluorescence spectroscopy, fluorescence microscopy, and transmission electron microscopy (TEM). Then, we revealed the difference in the suppressive effect on the FUS aggregation between FUS-binding non-coding RNA and irrelevant RNA.

## Results

### Shear stress caused by pipetting can induce FUS aggregation

**Figure 1:**
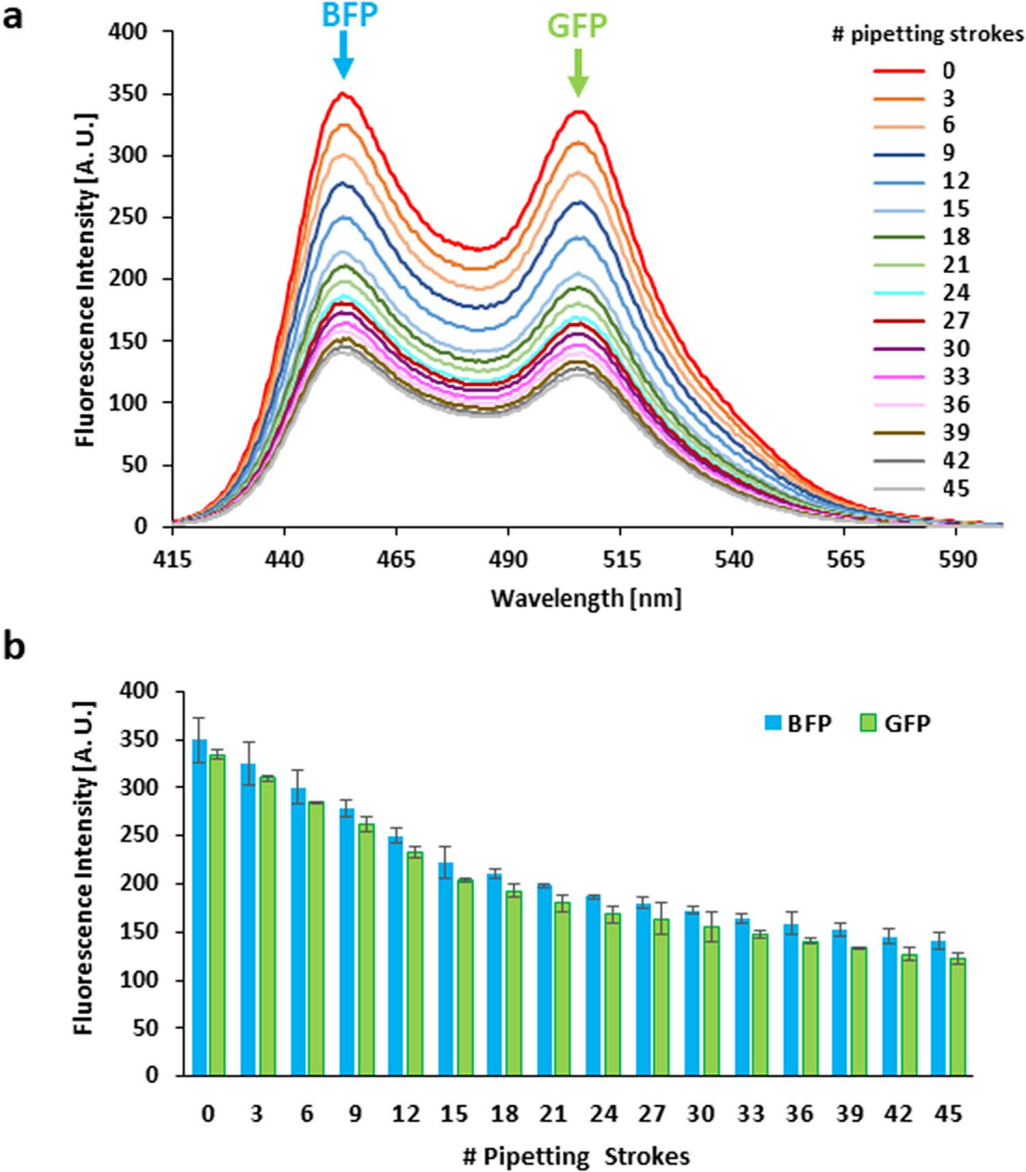
Effect of the number of pipetting strokes on the FUS fluorescence spectrum. (**a**) Overlaid fluorescence spectra of 100 nM FUS fusion protein (MBP-BFP-FUS-GFP-6xHis). The spectra were measured every 60 s, during which the sample (150 μL) was mixed by three strokes of pipetting (pipetting volume 140 μL). The total number of pipetting strokes is indicated on the right. (**b**) Bar graph showing the BFP (at 453 nm) and GFP (at 506 nm) fluorescence intensities of the fluorescence spectra shown in (**a**). The averages of two independent experiments ± standard deviation (SD) are shown.

We have been studying the mechanism of the transcription regulation of a cell cycle activator, the cyclin D1 gene (*CCND1*), by FUS in response to DNA damage^1,23–25^. We showed that a long non-coding RNA that is transcribed from the promoter region of *CCND1*, which was named promoter-associated non-coding RNA (pncRNA), can induce a conformational change of FUS. This conformational change enables FUS to interact with transcriptional coactivators, p300/CBP, and suppress their histone acetyl transferase activity. This leads to *CCND1* transcription suppression and subsequent cell cycle arrest, which may provide the time needed for DNA damage repair. We used FRET assaying to detect the effect of pncRNA on the conformational change of FUS^23^. FUS fusion protein with blue fluorescence protein (BFP) and green fluorescence protein (GFP) attached to its N- and C-termini, respectively, was used for the FRET assays. In that study, we noticed that upon sample pipetting, both the BFP and GFP fluorescence intensities decreased. To examine the effect of pipetting more quantitatively, 45 strokes of pipetting were applied to a FUS protein sample and fluorescence spectra were measured every three strokes of pipetting (the interval between measurements was set to 60 s) (Fig. 1). The sample volume was 150 μL and the pipetting volume was set to 140 μL. We supposed that the observed reduction in the fluorescence intensity for the entire wavelength range of 415–600 nm is caused by aggregation of FUS. Although the aggregates were not visible by eyes, they may precipitate or stay at the bottom of the cuvette and do not contribute to the fluorescence spectrum because the light does not pass the bottom of the cuvette. Then, as the concentration of the dissolved FUS is lower, the fluorescence intensity may reduce. In order to confirm this idea, the concentration of the protein in the supernatant of the sample was examined, as the aggregates are invisible by eyes and thus collecting and measuring the concentration of the protein in the precipitate were practically difficult. The concentration of the supernatant of the sample after 30 strokes of pipetting turned out to be lower than that of the sample without pipetting by ca. 40%. This reduction in concentration is qualitatively consistent with the reduction in the fluorescence intensity, supporting our idea.

Pipetting can subject protein molecules to shear stress due to the velocity gradient; shear stress is most prominent for molecules close to the surface of the pipette tip^26^. The unequal force distribution on a protein molecule might induce some conformational change that leads to aggregation. We checked whether aggregation could be induced just by incubating a sample in the fluorescence spectrophotometer cuvette without pipetting. No reduction in fluorescence intensity was observed when pipetting was not performed over the same time period (~ 15 min) (data not shown).

**Figure 2:**
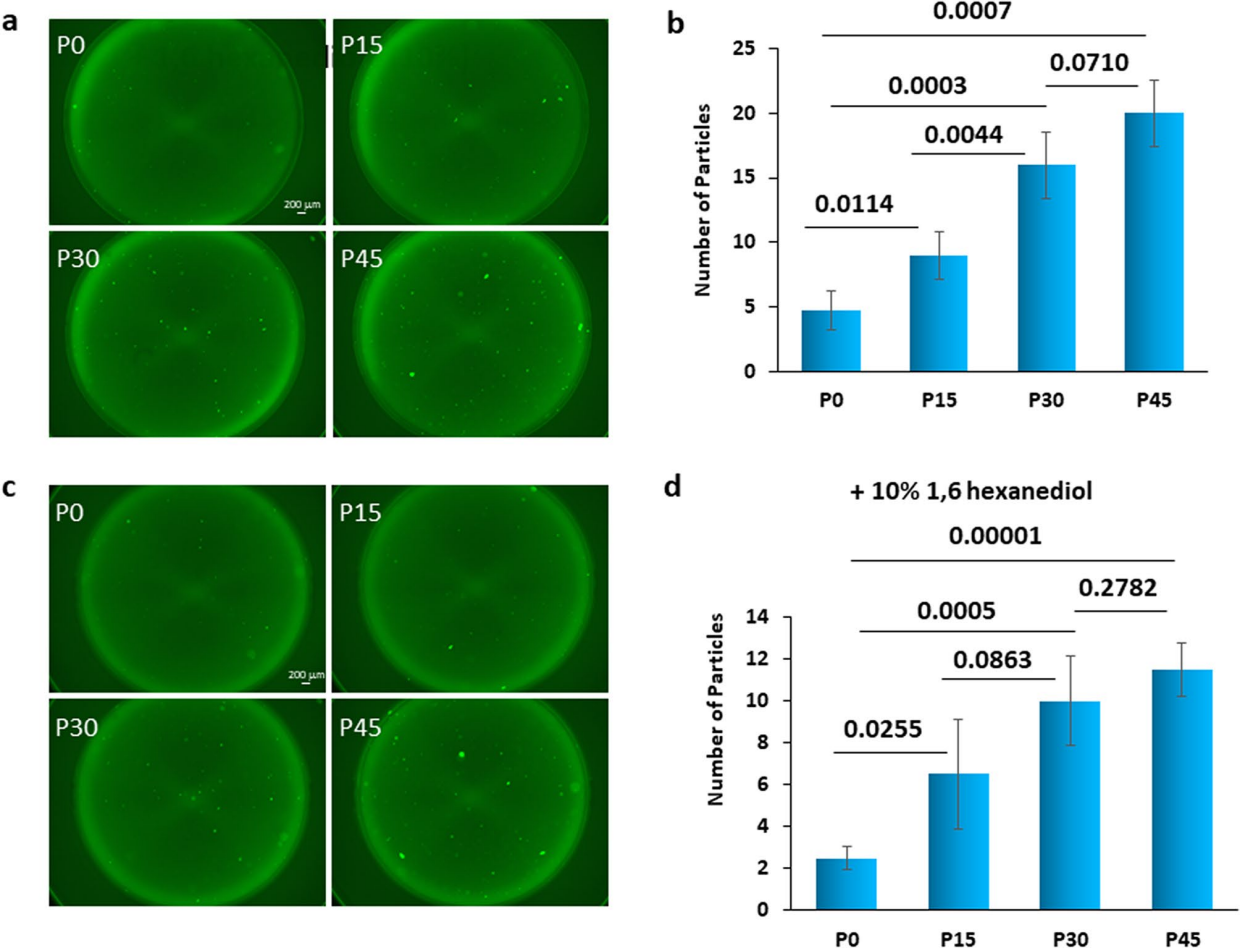
Fluorescence microscope images of FUS aggregates induced by pipetting. (**a**) Representative fluorescence microscope images of FUS fusion protein (Strep-GFP-FUS). The 100 nM Strep-GFP-FUS solution was subjected to pipetting 0, 15, 30, and 45 strokes (P0, P15, P30, and P45, respectively) before measurement. (**b**) A bar graph showing the number of particles > 0.002 mm^2^ observed in (**a**). The number of particles was determined with Fiji software. The bar graphs show the averages of three independent experiments ± standard deviation (SD). *p* values are indicated. (**c**) Images after addition of 10% 1,6-hexanediol, which is known to disrupt the liquid–liquid phase separation, to the samples shown in (**a**). (**d**) A bar graph showing the numbers of particles > 0.002 mm^2^ observed in (**c**).

To verify our assumption that the observed reduction in the fluorescence intensity of the FUS fusion protein is caused by FUS aggregation, the effect of pipetting on FUS was visualized by fluorescence microscopy. Another fusion protein, streptavidin recognition sequence (Strep)-GFP-FUS, was constructed in order to exclude the effects of MBP and BFP on FUS aggregation. We prepared four protein samples with different numbers of strokes of pipetting (0, 15, 30, and 45 strokes). Samples were prepared by diluting the stock protein solution 5 times with 3 gentle strokes of pipetting to get a thoroughly mixed protein solution of 100 nM. For the P0 sample, no further pipetting was applied. The P15, P30, and P45 samples underwent a further 15, 30, and 45 strokes of pipetting, respectively. All samples were examined by fluorescence microscopy. The number of particles larger than 0.002 mm^2^ was counted. More FUS particles were formed as the number of strokes of pipetting increased (Fig. 2a,b). Particles can be either droplets formed due to LLPS or aggregates. By using high magnification, the shapes of particles were examined. The shape of a droplet is known to be completely round^17^. The shapes of particles turned out to be mostly not round but irregular (Supplementary Fig. S2), indicating that most particles are not droplets formed due to LLPS but amorphous aggregates. We also examined the nature of the particles by using 1,6-hexanediol, which is known to dissolve liquid–liquid phase separated particles^27^. 10% 1,6-hexanediol was added to each sample, and then the particles were counted again. It was confirmed that the number of FUS particles that are resistant to 1,6-hexanediol treatment and thus are supposed to be not droplets but aggregates increased as the number of pipetting strokes increased (Fig. 2c,d). The sizes of the particles were also examined. The p45 sample has larger particles than the other samples. However, the number of small particles increased for the P45 sample as well. Thus, the average of the sizes of particles is nearly the same for all the samples.

**Figure 3:**
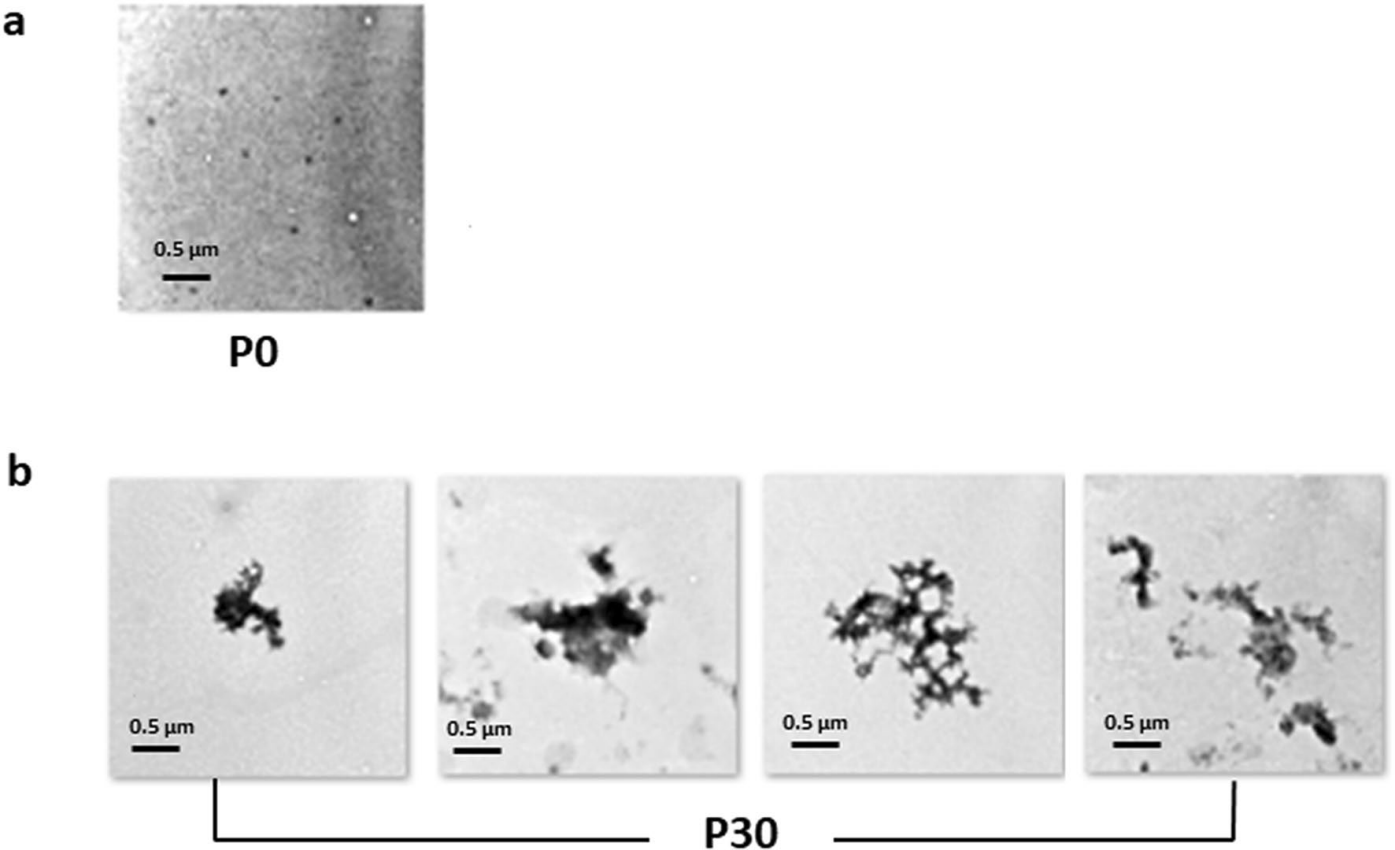
TEM images of FUS aggregates induced by pipetting. (**a**) A representative image of FUS fusion protein (MBP-BFP-FUS-GFP-6xHis) before pipetting (P0). (**b**) TEM images showing FUS aggregates formed after 30 strokes of pipetting (P30).

Next, we investigated the appearance of the pipetting-induced FUS aggregates by TEM to determine whether the formed aggregates are amorphous or take on a particular structure such as amyloid fibrils. A protein sample was examined before pipetting, the P0 sample (Fig. 3a), and after 30 strokes of pipetting, the P30 sample (Fig. 3b). For the P0 sample, aggregates were rarely found (Fig. 3a). It should be noted that the observed few dots are pores on a coated grid and not protein particles. However, for the P30 sample, many aggregates were found and they were mostly amorphous (Fig. 3b). Thus, although it might be difficult to decisively draw a conclusion that the FUS aggregates are formed by pipetting just from the observed reduction in the fluorescence intensity, analyses with two additional independent methods, fluorescence microscopy and TEM, solidly confirmed the conclusion.

### Sequence-specific suppression of FUS aggregation by non-coding RNAs

**Figure 4:**
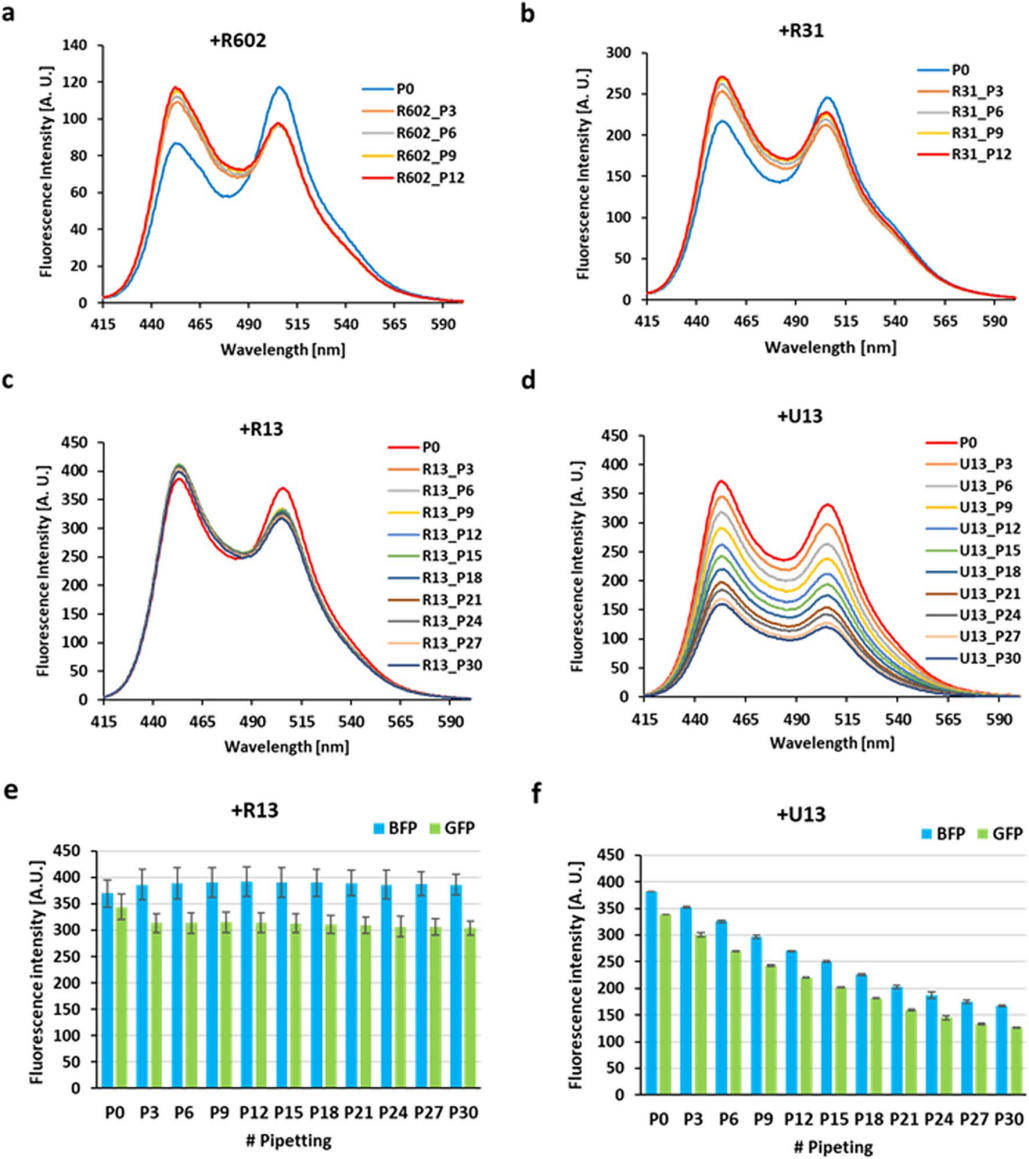
Sequence-dependent suppression of aggregation of FUS by RNA. (**a**–**c**,**e**) The fluorescence spectrum of 100 nM FUS fusion protein (MBP-BFP-FUS-GFP-6xHis) without pipetting is shown in blue (P0). An equimolar amount of either the full-length pncRNA (R602) (**a**), R31 (**b**), R13 (**c**), or U_13_ (**e**) was added to the protein solution. Then, fluorescence spectra were measured after every three stokes of pipetting: cumulative numbers of strokes are indicated, e.g., R602_P6 for six cumulative strokes of pipetting after the addition of R602 RNA. (**d**) A bar graph for BFP and GFP fluorescence intensities, at 453 and 506 nm, respectively, of the fluorescence spectra shown in (**c**). (**f**) The similar bar graph of the fluorescence spectra shown in (**e**).

In our previous studies, we showed that full-length (602 nucleotide residues) and fragments of pncRNA (Supplementary Fig. S1) can induce a conformational change of FUS^23,24^. Interestingly, this time we found that the reduction in the fluorescence intensity caused by shear stress on pipetting was not observed when full-length pncRNA was added to the FUS fusion protein (Fig. 4a). When the full-length pncRNA was added to FUS with a subsequent three strokes of pipetting to ensure thorough mixing, the pattern of the fluorescence spectrum changed due to the conformational change of FUS induced by pncRNA; the fluorescence intensity at 453 nm increased, while that at 506 nm decreased (Fig. 4a). We had already revealed that this spectrum change reflects the compact-to-extended conformational change of FUS with pncRNA on the basis of FRET and HS-AFM analyses^23,24^. When further strokes of pipetting were applied, however, the spectrum rarely changed, if any (Fig. 4a). The decrease in the fluorescence intensity caused by pipetting for all wavelengths, which was observed in the absence of RNA (Fig. 1a), was not seen. This observation indicates that pncRNA can protect FUS from aggregation caused by the shear stress of pipetting.

Previously, we examined the extent of the conformational change of FUS caused by pncRNA and its fragments on the basis of the change in FRET efficiency (Δ*E*), where *E* = I_GFP_/I_GFB_ + I_BFP,_ and I_BFP_ and I_GFP_ are the fluorescence intensities at 453 nm and 506 nm, respectively. As the 31-mer fragment of pncRNA (R31, see Supplementary Fig. S1) was revealed to be critical for binding to FUS^25^, R31 and shorter fragments of it were examined. Then, we found that the extent of the conformational change of FUS is greater for full-length pncRNA, R31, R19, and R13 than for R10, R7, R5, and R4 (see Supplementary Fig. S1 for sequences)^23^. That is, R13 is a minimum fragment to cause pronounced conformational change of FUS on binding. Here, addition of either R31 or R13 turned out to prevent aggregation of FUS (Fig. 4b–d) as well as that of full-length pncRNA. The decrease in the fluorescence intensity caused by pipetting for all wavelengths was not seen. Then, we examined the suppressive effect of a non-specific counterpart of R13, U_13_ comprising thirteen uracil residues. It was found that U_13_ cannot protect FUS from aggregation by pipetting. The fluorescence intensity continued to decrease with increasing number of strokes of pipetting (Fig. 4e,f). This indicates that suppression of FUS aggregation by R13 is sequence-specific.

**Figure 5:**
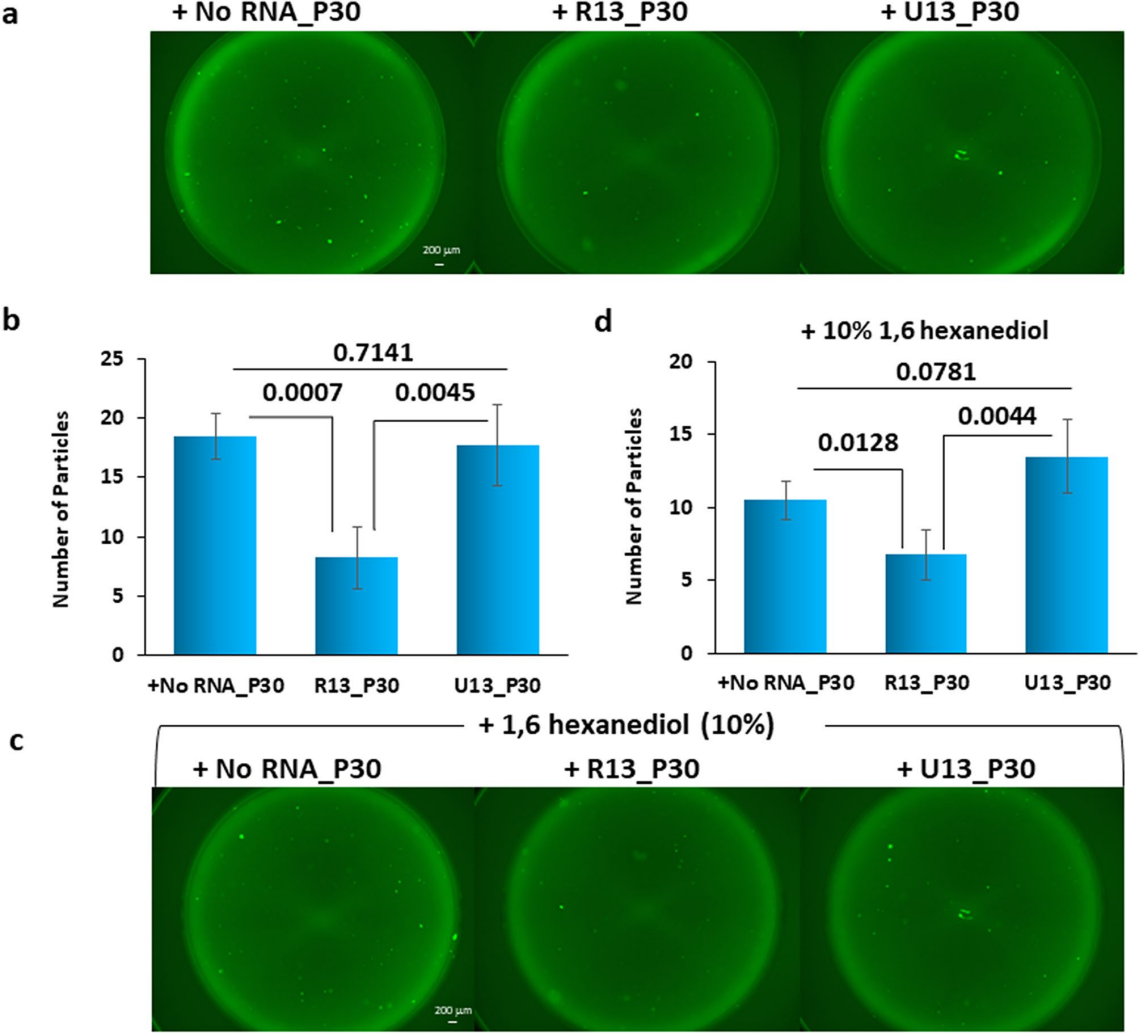
R13 represses the aggregation of FUS caused by pipetting while U_13_ cannot, as revealed by fluorescence microscope images. (**a**) Representative fluorescence microscope images of 100 nM FUS fusion protein (Strep-GFP-FUS) after the addition of an equimolar amount of either no RNA, R13 or U_13_ and subsequent application of 30 strokes of pipetting. (**b**) A bar graph showing the numbers of particles > 0.002 mm^2^. The bar graphs show the averages of 3 independent experiments ± SD. *p* values are indicated. (**c**) Images after addition of 10% 1,6-hexanediol to the samples shown in (**a**). (**d**) A bar graph showing the numbers of particles > 0.002 mm^2^ for images shown in (**c**).

**Figure 6:**
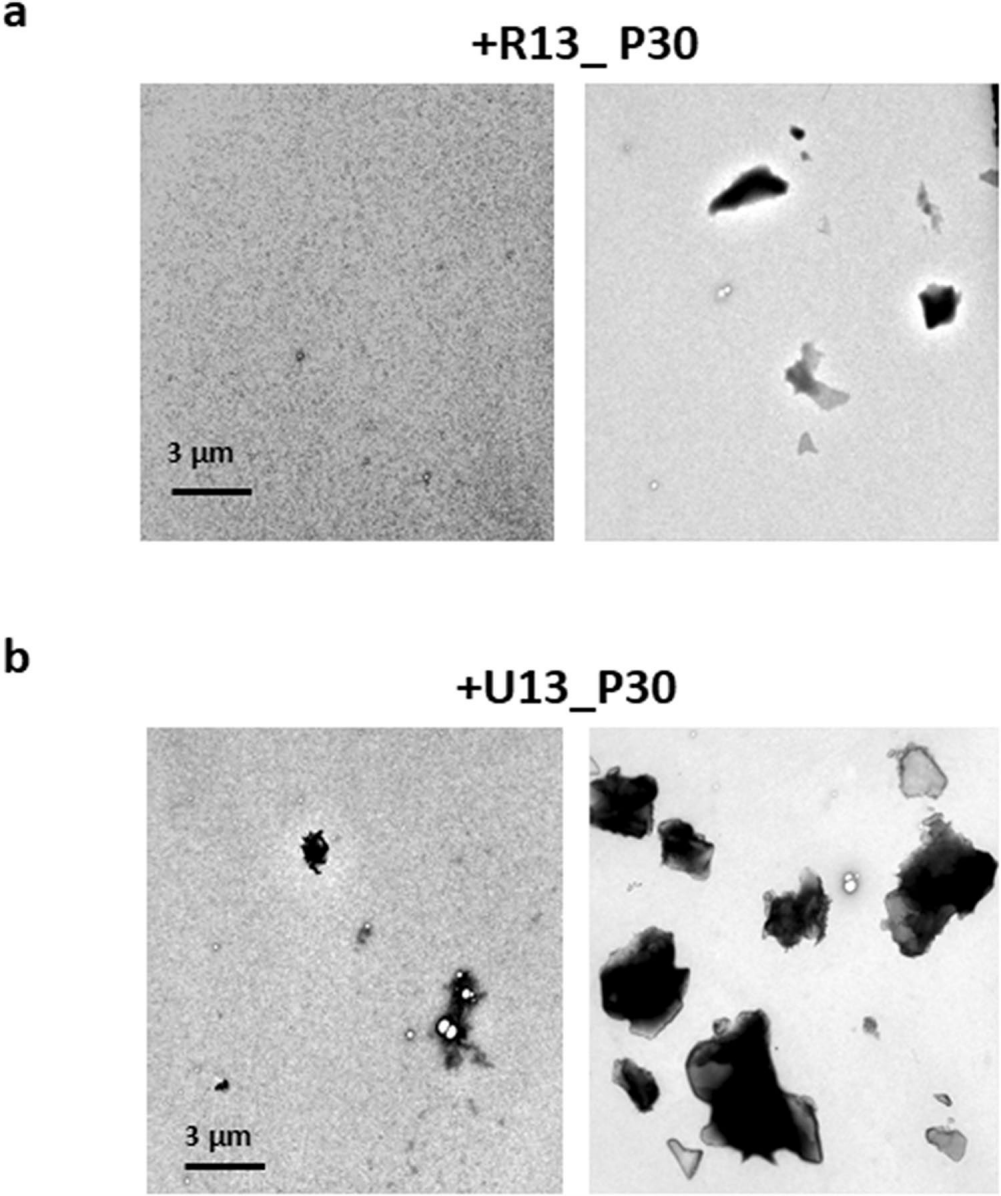
R13 represses the aggregation of FUS caused by pipetting while U_13_ cannot, as revealed by TEM. (**a**) Representative TEM images of FUS fusion protein (MBP-BFP-FUS-GFP-6xHis) after the addition of an equimolar amount of R13 and subsequent application of 30 strokes of pipetting. (**b**) Representative TEM images of FUS protein after the addition of an equimolar amount of U_13_ and subsequent application of 30 strokes of pipetting.

We further confirmed the difference between R13 and U_13_ as to the suppression of FUS aggregation by fluorescence microscopy (Fig. 5). Thirty strokes of pipetting were applied for 100 nM Strep-GFP-FUS protein in the absence of RNA, or in the presence of either R13 or U_13_. Samples were examined by fluorescence microscopy (Fig. 5a), and particles larger than 0.002 mm^2^ were counted (Fig. 5b). Then, phase separated particles were dissolved by adding 10% 1,6-hexanediol to each well (Fig. 5c), and the particles were counted again, only FUS aggregates being counted (Fig. 5d). This procedure showed that FUS aggregates were significantly less in the presence of R13 than in the presence of U_13_. This result confirmed that the suppression of FUS aggregation by R13 is sequence-specific. Finally, we confirmed the difference between specific and non-specific RNA on the suppression of FUS aggregation by TEM imaging (Fig. 6). In the presence of R13, only a few small aggregates were observed after applying 30 strokes of pipetting (Fig. 6a). On the other hand, many aggregates of various sizes were observed after applying 30 strokes of pipetting in the presence of U_13_ (Fig. 6b).

### Salt effect on FUS aggregation

**Figure 7:**
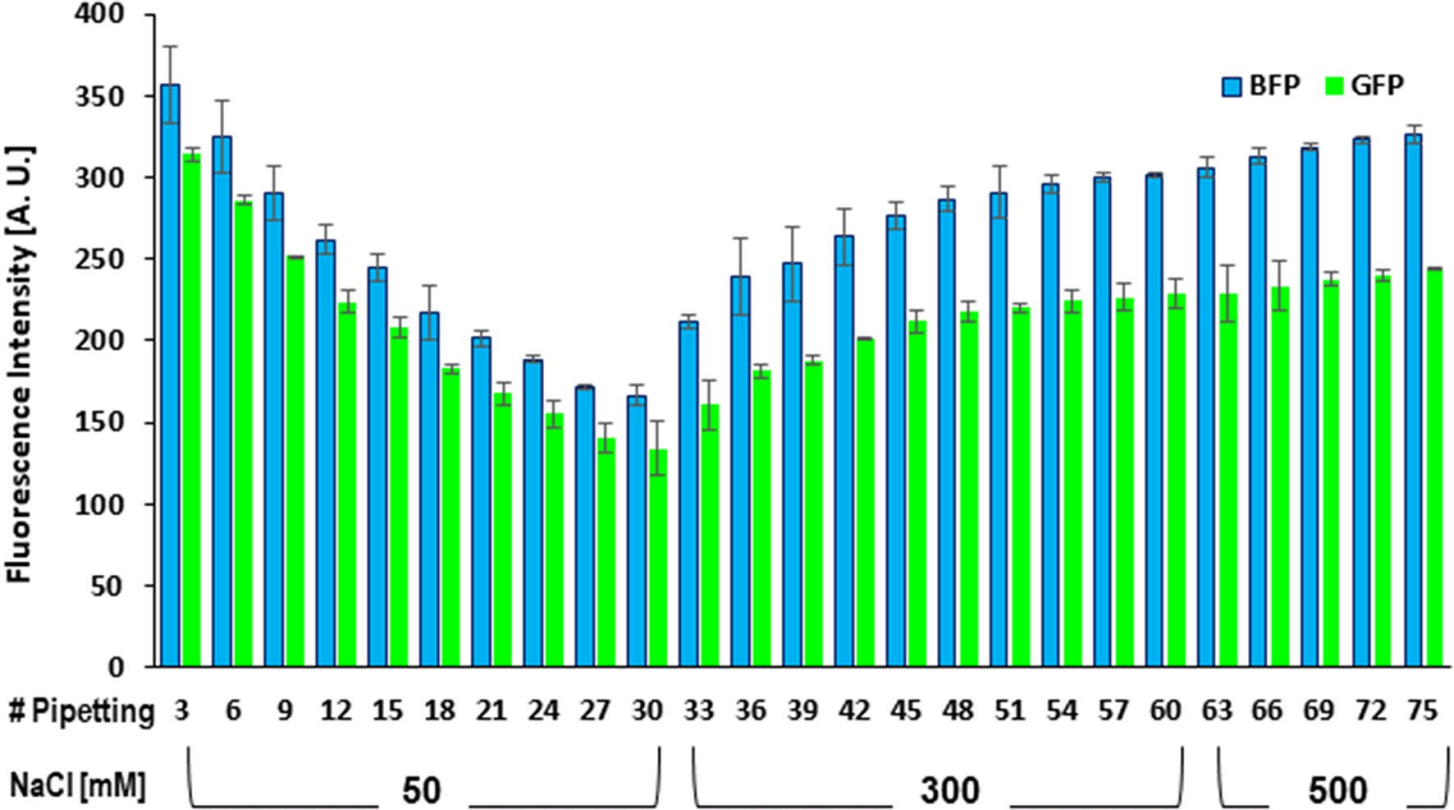
The effect of NaCl on FUS aggregation caused by pipetting. A fluorescence spectrum of 100 nM FUS fusion protein (MBP-BFP-FUS-GFP-6xHis) was measured at an initial NaCl concentration of 50 mM. Then, spectra were recorded after every three strokes of pipetting until the cumulative stroke number of 30. After that, the NaCl concentration was increased to 300 mM. Then, a total number of thirty stokes of pipetting was applied in the same way. After that, NaCl was further increased to 500 mM. Then, a total number of fifteen stokes of pipetting was applied similarly. Blue and green bars represent the BFP and GFP fluorescence intensities, respectively. The averages of two independent experiments ± standard deviation (SD) are shown.

Next, the effect of salt on FUS aggregation induced by pipetting was examined. Thirty strokes of pipetting were applied to 100 nM FUS fusion protein at 50 mM NaCl concentration to induce FUS aggregation. The reduction in fluorescence intensity due to aggregation was more when the number of pipetting strokes increased (Fig. 7). Then, the NaCl concentration was raised to 300 mM and an additional 30 strokes of pipetting were applied. In contrast to the situation with 50 mM NaCl, the fluorescence intensity was gradually restored as the number of pipetting strokes increased. Then, the NaCl concentration was further raised to 500 mM with an additional 15 strokes of pipetting. The fluorescence intensity basically remained the same in this case.

## Discussion

MBP-BFP-FUS-GFP-6xHis was used for FRET assaying and TEM imaging, while strep-GFP-FUS was used for fluorescence microscopy. The aggregation was found on pipetting for both MBP-BFP-FUS-GFP-6xHis and strep-GFP-FUS. Thus, it is not likely that either MBP or BFP is the origin of the aggregation. Additionally, it was reported that GFP does not aggregate on shearing^28^. Therefore, it is suggested strongly that FUS is the origin of the aggregation.

In this study, we demonstrated that the usual manner of pipetting can induce aggregation of FUS protein. FUS aggregates were visualized by TEM as being amorphous. The more the number of strokes of pipetting is, the more the number of aggregates is. We assumed that the shear stress caused by pipetting is a driving force for the formation of aggregates. The shear stress on the protein structure and the subsequent induction of aggregation such as amyloid fibril formation have been studied for decades^26,29–32^. Regarding the mechanism of shear stress-induced protein aggregation, there is a general consensus that mechanical perturbation of a protein molecule often results in structural destabilization of the native conformation, leading to the exposure of sequestered hydrophobic residues to the surrounding medium. Solvent-exposed hydrophobic groups become nucleated via hydrophobic interactions and subsequently aggregate^26^. Shear stress has been reported to induce fibril aggregation of the whey protein beta-lactoglobulin^29,33^. A rheo NMR study of superoxide dismutase 1 (SOD1) showed that shear stress can induce the formation of amyloid fibrils from SOD1 monomers, while under static conditions there is no change in the monomer state^34^. Therefore, it is possible that shear stress caused by pipetting induced the transition to the amorphous aggregate state.

The formation of aggregates was found at a low FUS concentration, even at 100 nM. This is in contrast to the situation that a higher FUS concentration, 1–5 μM, is usually needed for the formation of droplets due to LLPS^18,20,35^. The decrease in the fluorescence intensity of the FUS fusion protein was observed as an instant response to the pipetting, occurring in less than 60 s (Fig. 1). This indicates that FUS aggregates were instantly formed by shear stress caused by pipetting. This is also in contrast to the situation that a longer incubation time, minutes to an hour, is usually needed for the formation of droplets due to LLPS^35,36^.

Recently, shear-mediated formation of solid fibers of FUS was reported^20^. It was suggested that backbone-backbone hydrogen bonding constraints are a determining factor in governing the transition. A difference is noted that amorphous aggregates were formed in our case, while fibers were formed in that study. Shear stress was applied to FUS at a rather low concentration of 100 nM in our case, while it was applied to a LLPS form of FUS in that study. This may be related to the formation of different species, amorphous aggregates or fibers.

The number of liquid droplets might be obtained by calculating the difference in the number of particles between Fig. 2b,d, because it is supposed that the number of particles of Fig. 2b is a sum of the number of aggregates and that of liquid droplets while the number of particles of Fig. 2d is the number of aggregates as liquid droplets are assumed to be dissolved by 1,6-hexandiol. However, we decided not to evaluate the number of liquid droplets, because the difference in the number of particles between Fig. 2b,d is too small and thus not reliable to evaluate. We decided to discuss just the number of aggregates, because the corresponding numbers are relatively large and thus more reliable to evaluate.

The biological significance of the conformational change caused by shear stress has been suggested. In vivo, blood flow in narrow capillaries can induce considerable shear stress in the circulatory system^37^. The biological significance of shear stress can be represented by the conformational change of a human blood plasma protein, von Willebrand (vWF), which has an important function in coagulation. It was suggested that high shear stress at the site of bleeding injury could induce a structural change in vWF, which is critical for platelet adhesion and thrombus formation at the wound site^38^. Moreover, at the cellular level, the velocity of organelles inside cells is not uniform. Therefore, a velocity gradient arises, which in turn creates shear stress on proteins inside the cells.

It was reported that RNA regulates the phase behavior of FUS^18^. Low RNA/FUS ratios promote the formation of droplets due to LLPS, whereas high ratios prevent the droplet formation in vitro. Reduction of nuclear RNA levels or genetic ablation of RNA binding causes the formation of cytotoxic solid-like assemblies in cells^18^. So far, on the other hand, an effect of RNA on the shear-mediated formation of solid fibers of FUS from the liquid–liquid phase separated form has not been reported in the literature. Here, we showed that full-length pncRNA and its fragments, R31 and R13, can protect FUS from aggregation caused by shear stress on pipetting (Figs. 4, 5, 6). We also showed that U_13_ cannot protect FUS from aggregation although its length is the same as that of R13. That is to say, the suppressive effect of RNA is sequence-dependent. This is one of the limited cases where the RNA sequence-specific suppression of aggregation of a protein was clearly revealed.

It was reported that the prion-like domain (residues 1–239) comprising the LC domain and a part of the first RGG domain in the N-terminal region and the second RGG domain (374–422) in the C-terminal region are essential for aggregation^39^. Previously, we revealed that pncRNA and its fragments bind to the C-terminal region of FUS comprising the second RGG domain (374–422), a ZnF domain (423–453), and the third RGG domain (454–526)^25^. Therefore, there is the possibility that RNA bound to the C-terminal region of FUS masks the interface required for the formation of aggregates, resulting in the prevention of aggregate formation. It was also reported that the cation-π interaction between the N-terminal LC domain and C-terminal RGG domain is critical for LLPS of FUS^35,40^. Therefore, it is also likely that RNA bound to the C-terminal region of FUS neutralizes the cations and reduces the cation-π interaction. The reduction of the interaction may prevent FUS not only from LLPS but also from aggregation. Specific RNAs bind to FUS with higher affinity than non-specific ones. This can explain why the suppressive effect on aggregation of FUS is RNA sequence-dependent. It might also be the case that non-specific RNA does not necessarily bind to the interface needed for the formation of aggregates, resulting in a lower suppressive effect. The correlation between the extent of conformational change of FUS caused by each RNA, which was estimated by Δ*E*, and resistance to aggregation by each RNA may also imply that the FUS conformation induced by RNA is unfavorable for the formation of aggregates. It would be added that TEM images of aggregates of FUS in the presence of U_13_ looked different from those of FUS alone. This may be due to involvement of U_13_-bound FUS to some extent in aggregates in the former case.

It should be added that the FUS-RNA interaction was not likely to be affected by the presence of MBP, GFP, and BFP. MBP has no RNA binding activity^41^. Fusion of GFP to FUS is widely used for marking FUS even in the studies of the FUS-RNA interactions^18,42^, which indicates that GFP is not supposed to interact with RNA. Additionally, BFP whose sequence similarity with GFP is 93% is not supposed to interact with RNA, either.

The recovery of the florescence intensity of FUS on pipetting with 300 mM NaCl (Fig. 7) indicated that the FUS aggregates can be dissolved, at least partially, at 300 mM NaCl. This suggests that the electrostatic interaction and/or the cation-π interaction contribute to the formation of the aggregates and that weakening of the interaction(s) at higher NaCl concentration results in the partial dissolution of the aggregates. Previously, it was reported that LLPS of FUS is not affected by raising of the NaCl concentration from 50 to 150 mM but that LLPS is significantly reduced at 300 mM NaCl^43^. The suppressive effect of NaCl is suggested to be common to LLPS and aggregation of FUS.

In conclusion, we found that the shear stress caused by pipetting instantly induces the transition of FUS to amorphous aggregates even at low FUS concentration. The non-coding RNA we previously identified, pncRNA, can suppress this transition in a sequence-dependent manner. Our finding might serve for the development of therapies for neurodegenerative diseases by using RNA as aggregation inhibitors.

## Materials and methods

### Protein preparation

MBP-BFP-FUS-GFP-6xHis protein was expressed and purified as described previously^23,24^. Briefly, the fusion protein was expressed in BL21 Gold (DE3) *Escherichia coli* cells. The protein was induced by the addition of 0.1 mM isopropylthio-β-d-galactopyranoside (IPTG) for 20 h at 20 °C. Cell pellets were sonicated in lysis buffer comprising 50 mM Tris–HCl (pH 7.6), 25 mM glucose, 1% CHAPS, 10 mM benzamidine, 5 U/mL DNase I, 1 mg/L RNase, and 0.2 g/L lysozyme. The supernatants were purified by nickel-affinity column chromatography using Ni-sepharose beads (GE Healthcare Bio-Sciences), followed by size exclusion chromatography (SEC) using a HiloadTM 16/60 SuperdexTM 200 prep grade column (GE Life Sciences). Protein was stored at 4 °C. 5 mM fresh dithiothreitol (DTT) was added to a purified fusion protein solution on the same day as the experiment. The protein sample was diluted five times to obtain ~ 100 nM FUS fusion protein in 10 mM Tris–HCl (pH 7.6), 5 mM glucose, 0.2% CHAPS, and 50 mM NaCl. This fusion protein was used for FRET assaying and TEM imaging.

Strep-GFP-FUS protein was expressed using BL21. The cells were collected and frozen in −80 °C overnight, and then resuspended in WCE buffer (25 mM HEPES (pH 7.9), 150 mM NaCl, 1.5 mM MgCl_2_, 0.2 mM EDTA, 0.05% Triton X-100, and 10% glycerol). Cells were lysed by sonication on ice. The cell lysates were centrifuged at 110,000*g* for 40 min. The supernatants were purified on a Strep-Tactin Sepharose column (gravity flow, IBA Lifesciences). The column was washed with wash buffer (100 mM Tris–HCl (pH 8.0) 150 mM NaCl, and 1 mM EDTA), and protein was eluted with elution buffer (100 mM Tris–HCl (pH 8.0), 300 mM NaCl, 1 mM EDTA, and 2.5 mM desthiobiotin). The protein was concentrated with Amicon ultra-0.5 centrifugal filters to a final concentration of around 5 μM. For the aggregation assays, 100 nM sample was dissolved in 10 mM Tris–HCl (pH 7.5) and 50 mM NaCl. This fusion protein was used for fluorescence microscopy.

### Fluorescence spectroscopy

A final concentration of 100 nM FUS in 10 mM Tris–HCl (pH 7.6), 0.2% CHAPS, 5 mM glucose, 50 mM NaCl, and 1 mM DTT was prepared in a total volume of 150 µL by diluting the FUS stock solution (50 mM Tris–HCl (pH 7.6), 1% CHAPS, 25 mM glucose, 250 mM NaCl, and 5 mM DTT). For the experiments in the presence of RNA, an equimolar amount of RNA was added to the FUS solution. Fluorescence spectra were collected with a steady-state photon counting spectrofluorometer (JASCO FP-8500 spectrometer, Japan Spectroscopic Co.) using a standard quartz cuvette with an optical path length of 1 cm. The excitation wavelength was 402 nm. The spectra slit width of 5 nm was used for excitation and emission with an integration time of 1 nm/s from 415 to 650 nm. All the measurements were carried out at 25 °C. A blank was measured and subtracted from all the spectra. Data were processed using a JASCO Spectra Manager of the FP-8000 series.

Pipetting was carried out in a cuvette using a pipette tip whose point orifice and root diameters, and length are ca. 0.5 mm, 3.9 mm, and 36 mm, respectively. Sucking up was done in ca. 0.5 s, followed by extruding in ca. 0.5 s, one stroke of pipetting being accomplished every one second. The concentration of the sample after pipetting was measured as follows; after spinning the sample solution, the supernatant was collected and its concentration was measured on the basis of UV absorbance.

### Fluorescence microscopy

100 µL of 100 nM protein was placed in a 96-well plate and visualized under different pipetting conditions. GFP fluorescence was observed under a fluorescence microscope (Keyence BZ-X710). Images were converted to black and white. Fiji software (RRID: SCR_002285) was used to set particle size parameters to count particles > 0.002 mm^2^ to exclude background noise. To distinguish between LLPS particles and aggregates, 10% 1,6-hexanediol was added to each well, with which LLPS particles are dissolved, while aggregates are preserved. *p* values were calculated by two-tailed T test using T.TEST function of Microsoft Office Excel (2019).

### Transmission electron microscopy

A sample solution was diluted tenfold up to 10 nM and then spotted onto a collodion‐coated copper grid (Nisshin EM Co., Tokyo, Japan). After 1 min, the remaining solution was removed with filter paper and 5 μL of 1% (w/w) phosphotungstic acid (PTA) was spotted onto a collodion‐coated copper grid. The solution was removed immediately with filter paper. Then, the grid was washed with 5 μL pure water to remove excess PTA. After 1 min, the remaining solution was removed in the same manner. Images were obtained using a H‐7650 TEM (Hitachi, Tokyo, Japan) operating at 80 kV.

## Supplementary Information

Supplementary Information 1.
