# Supplementary material for: Non-coding RNA suppresses FUS aggregation caused by mechanistic shear stress on pipetting in a sequence-dependent manner
Source: Sci Rep. 2021 May 4;11:9523. doi: 10.1038/s41598-021-89075-w (PMC8096841; doi:10.1038/s41598-021-89075-w)
Supplement: Supplementary file 1 — Supplementary Information 1. [file 41598_2021_89075_MOESM1_ESM.pdf]

a

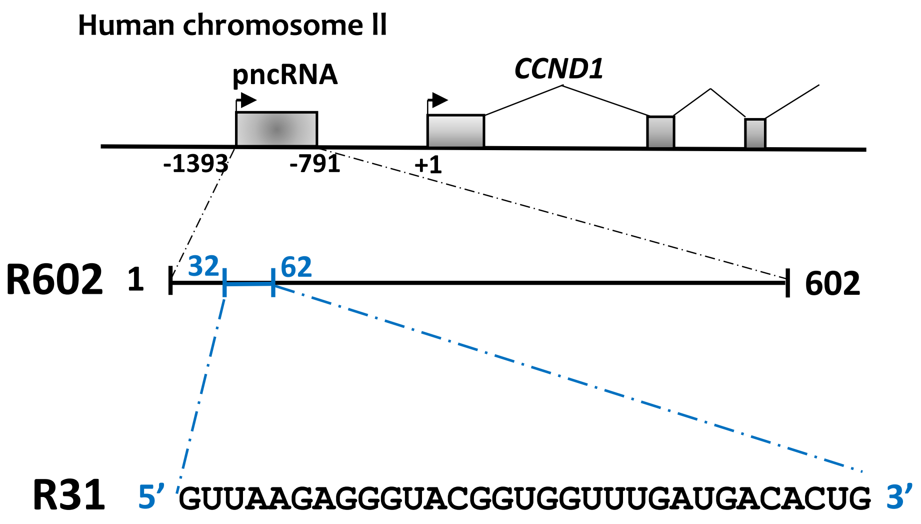

b

R31	GUUAAGAGGGUACGGUGGUUUGAUGACACUG
R19	CGGUGGUUUGAUGACACUG
R13	GUUAAGAGGGUAC
R10	GGUUUGAUGA
R7	GUUAAGA
R5	AGGGU
R4	GGGU

Supplementary Figure S1. Illustration of the full-length promoter-associated non-coding RNA (pncRNA) and its shorter fragments referred to in this study.

(a) Schematic representation of the localization of full-length pncRNA (R602) and its critical short fragment, R31 (1). **(b)** The names and sequences of fragments of R31.

1. Yoneda, R. *et al.* The binding specificity of Translocated in LipoSarcoma/FUsed in Sarcoma with lncRNA transcribed from the promoter region of cyclin D1. *Cell Biosci.* **6**, 4 (2016).

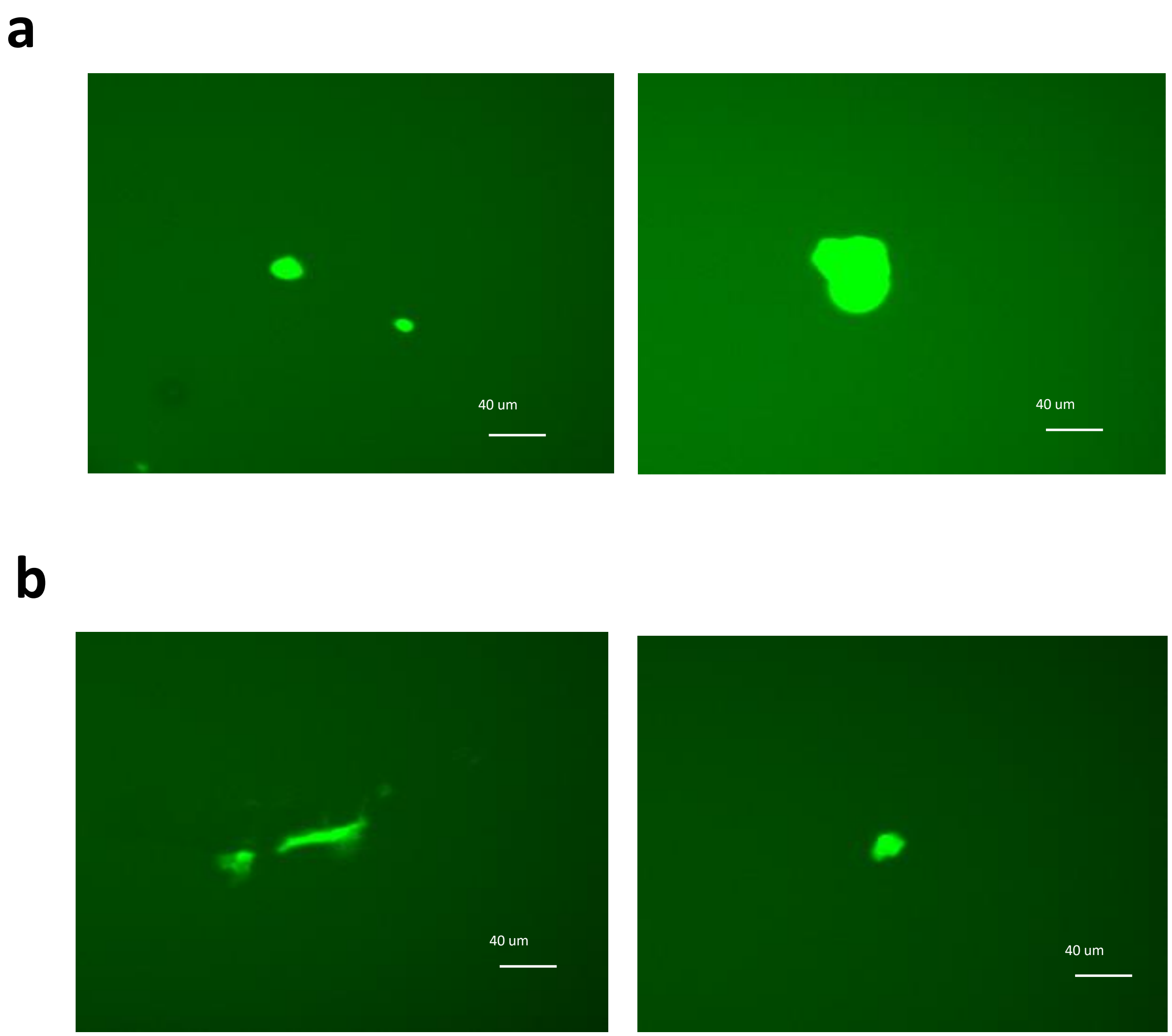

Supplementary Figure S2. Images of FUS particles by fluorescence microscope with higher magnification. Representative images of the FUS fusion protein (Strep-GFP-FUS) for the sample without pipetting (a) or with 30 strokes of pipetting (b).
